# Progress in Psychiatry

**Published:** 1900-08-11

**Authors:** 


### Progress in Psychiatry.

(*Continued from page* 305.)

**Criminality and Degeneracy.**—In referring to the stigmata of degeneracy in a previous chapter (p. 173 *ante*), it was stated briefly that "among lunatics, criminals, paranoiacs, and congenital imbeciles about 30 per cent. or more will be found to exhibit stigmata of degeneracy in unmistakable form." While, however, some authors have exaggerated the importance and significance of these stigmata, others have attempted to minimise them or to explain them away altogether. The bare facts, however, as ascertained by competent neurological and anthropological observers may be here dealt with, and their significance pointed out as occasion arises. Thus, projecting superciliary ridges on the head or skull were found in 46 per cent. of criminals, the material being 609 crania carefully examined.[47] Abnormal closures of the cranial sutures (syostoses) were similarly found in 43 per cent. of the same crania. As regards formation and shape of the skull it appeared further that 32 per cent. of the crania were microcephalic, 25 per cent. were plagiocephalic (in the sense already explained in the section on anatomical stigmata of degeneracy), and 10 per cent. were stenocephalic. Among the rarer anomalies of cranial configuration were trochocephalic heads (9 per cent.), and oxycephalic heads (4 per cent.).

From the studies made on 236 crania of criminals by Corre, Benedikt, Flesch, Roncoroni, and Ardu we get the following data: 36 per cent. showed osteo-sclerosis (with more or less abnormal union of cranial sutures); 19 per cent. showing marked obliquity of the orbit, tending to asymmetry of the two halves of the face; 19 per cent. showed enormous development of the upper jaw, and 69 per cent. showed prognatrism; 10 per cent. showed a retreating chin, a characteristic often associated with intellectual or moral imbecility. Lombroso concludes, on what seems to be excellent and sufficient grounds of observation, that "cranial and facial asymmetry prevails among criminals to an extent four to seven times as great as in the normal population."

Regarding the relative numerical prevalence of stigmata of degeneracy in various classes of the population, the following table from Lombroso[48] is very instructive:—

Signs of Degeneracy in 25,000 People.

| | 5 or more stigmata. | 3 or more stigmata. |
|---|---|---|
| Normals ... | 4 per cent. ... | 24 per cent. |
| Epileptics ... | 29 ,, ... | 55 ,, |
| Delinquents ... | 27½ ,, ... | 33½ ,, |
| Insane ... ... | 25⅓ ,, ... | 55 ,, |

A careful study of this valuable and instructive table shows the much greater relative predominance in criminals of stigmata of degeneracy when several stigmata—*e.g.*, five or more—are considered, so that their cumulative value and significance become undoubted. We then find that delinquents or criminals exhibit them seven times as often as normals, and that they are in this respect closely allied to the epileptic and the insane. The latest studies of Frigierio, Curdali, Bianchi, and Lombroso upon epileptics and the morally insane show so many analogies between the two as to leave no doubt as to their common origin and nature.

In estimating the locus of the criminal in the social and moral scale it is important to bear in mind the facilities or opportunities which a certain social environment may offer towards the development (or repression) of his criminal instincts. The criminal, unlike the normal man, is so much inclined to orgies of lust and drink that it is hard to ascertain whether these play a casual or a causal part in criminality. "After the pleasures of vengeance and of satisfied vanity," says Lombroso, "the criminal finds no amusement to equal that afforded by wine and play . . . . Alcohol is an instrument and a reason for crime, because some delinquents commit crime in order to get drink, or, again, because by drinking cowards acquire courage and recklessness sufficient to the performance of crime, as well as a certain justification (extenuation) in the eyes of the law. Moreover, in low quarters the saloons are the resorts of criminals who meet their accomplices, and who often deposit with the landlords or proprietors their illegitimate gains."

Dr. David Nicolson, late medical superintendent of the Asylum for Criminal Lunatics at Broadmoor, believes that the criminal instinct may for purposes of study and classification be regarded as threefold, viz., as composed of instincts towards thieving, towards violence against the person or destructiveness towards property, and towards sexual offences. "This threefold (criminal-like) instinct is a universal birthright which man shares with all animals, and it would be our natural characteristic throughout life were it not for the gradual development in us of certain higher (and inhibitory) intellectual and volitional processes, such as prudence, reflection, and a sense of moral duty." In proportion as this normal development is prevented or stifled—whether from congenital brain defect, or from lack of proper education and training—so there is a risk of the individual lapsing into criminal proclivities, or into actual crime. And this risk becomes intensified if the parents and their associates are people of vicious or drunken habits.

As regards the crimes committed by criminal lunatics, it appears, according to the same authority, that "96 per cent. of the crimes of criminal lunatics are crimes of violence," usually referable to mental exaltation (mania), or mental depression (melancholia), and often accompanied by delusions. Less frequently crimes may be due to epilepsy. It is also, according to French and Italian observers, largely a matter of temperament as to the exact nature of the crimes committed by criminals whether sane or insane.[49] The criminal like the degenerate is dangerous to society, and both of them like the lunatic require separation from the community at large for their own sake and for the protection of society. The existing provision appears in the form of asylums, prisons, and reformatories. The chronic inebriate belongs to a similar dangerous class, and is by the enactment of recent legislation similarly amenable to sequestration. And the prevalence of sexual and homo-sexual offenders and other degenerates in the community has awakened the minds of physicians and legislators to the needs of special provision for this class of offenders. The presence of certifiable and dangerous lunatics—in small numbers it is true, but nevertheless to a dangerous extent—in our midst is a fact which requires to be impressed on the minds of rulers and local authorities. The problem of the unrestrained lunatic in the community at large occasionally assumes prominence in the lay press, only to subside into oblivion soon after. "That our criminal lunacy is largely preventable," writes Dr. David Nicolson,[50] "there is no doubt, and the responsibility for its recurrence is often traceable to (*a*) *delay in certifying* an insane person . . . or (*b*) *dilatoriness in removal* of certified lunatics to an asylum, or to (*c*) *premature discharge* from an asylum. The number of murders and attempted murders that result from the condition of things here mentioned is very considerable. There are walking about in our midst plenty of potential criminal lunatics whose insanity indeed is not unrecognised; nobody knows when it may be his turn to enact the part of victim in a realistic drama of blood."

[47] The figures in this paragraph are from Lombroso's article on Criminology in the Twentieth Century Practice of Medicine, vol. xii. [48] Loc. cit., p. 581. [49] Lancet, April 7, 1900. [50] Clifford Allbutt's System of Medicine, vol. viii.

